# Comparative toxicity of three variant oils and their nanoemulsions on the brown dog tick *Rhipicephalus sanguineus*

**DOI:** 10.1038/s41598-024-77402-w

**Published:** 2024-11-07

**Authors:** Hoda S.M. Abdel-Ghany, Fathalla Ayoob, Sobhy Abdel-Shafy, Ahmed A. F. Soliman, Mohamed A. Gebely, Mai Abuowarda

**Affiliations:** 1 Department of Parasitology and Animal Diseases, Veterinary Research Institute, National Research Centre, Dokki, Giza Egypt; 2 Ticks and Tick-Borne Diseases Research Unit, Veterinary Research Institute, National Research Centre, Dokki, 12622 Giza Egypt; 3 Department of Tanning Materials and Leather Technology, Chemical Industries Research Institute, National Research Centre, Dokki, Giza Egypt; 4 Department of Pharmacognosy, Pharmaceutical and Drug Industries Research Institute, National Research Centre, Dokki, Giza Egypt; 5 Department of Parasitology, Faculty of Veterinary Medicine, Cairo University, PO: 12211, Giza, Egypt; 6 Department of Parasitology of Veterinary Medicine, Cairo University, Cairo, Egypt

**Keywords:** *Rhipicephalus sanguineus (s.l.)*, Dogs, Acaricide, Nanoemulsion, TEM, Fibroblast cell, Microbiology, Medical research

## Abstract

Tick control mainly depends on using chemical acaricides that have led to the emergence of resistant tick populations along with environmental hazards. Natural alternatives including essential oils are now widely used to avoid the undesirable effects of chemicals on human, animals and environment. In this study, three commercial oils (myrrh, patchouli, and cypress) and their nanoemulsions (NEs) were tested against *Rhipicephalus sanguineus sensu lato* unfed adults. Transmission electron microscopy (TEM) and dynamic light scattering (DLS) were used to characterize the nanoemulsions. Four concentrations with three replicates were used in the bioassay of oils and NEs against ticks using an adult immersion test. Furthermore, a toxicity study of the three oils and their NEs using normal fibroblast cells (BJ-1) was performed. TEM revealed particle size ranges from 29 to 211 nm with spherical droplets. The droplet size and polydispersity index were (29.30 nm & 0.367), (211.6 nm & 0.221) and (164.2 nm & 0.237) for myrrh NE, patchouli NE, and cypress NE, respectively. Myrrh and patchouli oils recorded high acaricidal activity followed by cypress oil with LC_50_ value 5 days after treatment of 9.01, 12.40, and 15.21%, respectively. The acaricidal efficacy of oil was potentiated in the NEs form which was proved by the reduction in LC_50_ values that were recorded 7 days after treatment 4.17, 8.57, and 5.04%, for myrrh, patchouli, and cypress NE, respectively. As an alternative for lab animals, we applied the cytotoxicity of myrrh, patchouli, and cypress on normal fibroblast cells. The oils showed low cytotoxic activity of 10.6, 21.5, and 23.6% for Myrrh, Patchouli, and Cypress, respectively. The nanoemulsion was revealed to be safe for normal cells up to a concentration of 0.62% (cytotoxicity%= 24.4, 34.4, and 16.3%, respectively), and the moderate cytotoxic effect appeared at around a concentration of 1.25% (cytotoxicity%= 42.2%, 57.0%, and 36.8% respectively). In conclusion, the three oils and their NEs have good acaricidal activity against *Rhipicephalus sanguineus* unfed adults. Further toxicity studies on mammals are needed to ensure the safe use of these formulations for the control of ticks on infested animals.

**Supplementary Information:**

The online version contains supplementary material available at 10.1038/s41598-024-77402-w.

## Introduction

*Rhipicephalus sanguineus* (Latreille, 1806) recognized as the brown dog tick is a worldwide tick that mainly parasitizes dogs and leads to skin damage, blood loss, and allergic reaction^1^. In addition, it acts as a vector for various pathogens belonging to protozoa, bacteria, viruses, and helminths that threaten animal and human health^2–6^.

The current control of ticks is mainly based on the application of chemical acaricides^7,8^. However, the indiscriminate and persistent use of these acaricides allows the emergence of many resistant tick species as well as environmental and public health hazards^9^. Consequently, alternatives should be found to be safer and more effective than these acaricides.

Oils derived from natural plants are considered a promising alternative as they contain different active constituents that have an effect against both susceptible and resistant ticks besides their fast biodegradability and being less toxic to mammals^10–12^. Studies performed on different classes of ticks using oils and plant extracts possessed 5-100% efficacy^13^. Although oils are seen to be a promising replacement, they have certain drawbacks including limited water solubility, high volatility, and a propensity for oxidation^14^. Therefore, nano-formulations of these oils in the form of nano-emulsions might be useful to overcome these problems as they improve oil stability, reduce particle size, control oil release, and increase therapeutic potency^15–17^.

Some studies recorded the acaricidal efficacy of oils against *R. sanguineus* ticks as *Lippia sidoides* essential oil on larvae and nymphs^18^; *Syzygium aromaticum* (L.) essential oil and eugenol on non-fed larvae^19^; and *Piper macedoi* essential oil on larvae^20^. Few studies evaluated the acaricidal activity of nanoformulations against *R. sanguineus.* Monteiro et al.^21^ evaluated thymol and eugenol microemulsion in vivo on dog tick *R. sanguineus* which was efficient for larvae and affected the reproductive efficiency of engorged females.

There are a few studies on the acaricidal efficacy of plant materials obtained from myrrh (*Commiphora myrrha*), patchouli (*Pogostemon cablin*) and cypress (*Cupressus sempervirens*) against either ticks or mites. The essential oil of myrrh *Co. myrrha* revealed acaricidal activity against the adult females of red spider mite *Tetranychus urticae* C. L. Koch, (1836) (Acari: Tetranychidae)^22^. A hexane extracts of the resin of *Co. holtziana* and *Co. myrrha* were evaluated against the cattle tick, *Boophilus microplus* (Canestrini, 1888) (Acari: Ixodidae), *Co. holtziana* exhibited a repellent effect while *Co. myrrha* was inactive^23^. The patchouli oil *Pogostemon cablin* inhibited the oviposition of *Tetranychus cinnabarinus* (Acari: Tetranychidae)^24^. The essential oil of cypress *Cu. sempervirens* revealed acaricidal activity against adults, larvae and eggs of the tick *Hyalomma scupense* Schulze, 1919 (Acari: Ixodidae)^25^. The acaricidal activity of these oils against the brown dog tick *Rhipicephalus sanguineus* was not evaluated before. The commercial form of these oils is cheap and available in local markets and can be used in the treatment of dogs infested by *R. sangiuneus* ticks. Moreover, these oils seem to be less harmful to human and the environment than chemical acaricides and already are used by humans.

Therefore, it was important to direct the aim of the present work to evaluate the acaricidal and in vitro cytotoxic efficacy of myrrh, patchouli, and cypress oils and their nanoemulsions against unfed adult of dog tick *R. sanguineus (s.l.)*.

## Materials and methods

### Unfed adult ticks

Fully engorged females and males of *R. sanguineus (s.l.)* were collected from infested dogs which were referred to a private clinic, in Giza, Egypt. Ticks were collected in a special glass tube containing strips of filter paper. Ticks’ morphological identification was performed according to the key of Walker et al.^26^. These females were incubated at 25 ± 1 °C and 75–80% relative humidity (RH) for oviposition. Using the capsule method, the unfed larvae were allowed to feed on healthy rabbits after hatching^27^, then collected and incubated for molting to nymphs. After molting, the unfed nymphs were allowed to feed on healthy rabbits to obtain fully engorged nymphs that were incubated at the same conditions mentioned above for molting to unfed adults which were used in the bioassay.

### Oils

The three oils; myrrh (*Commiphora myrrha*), patchouli (*Pogostemon cablin*), and cypress (*Cupressus sempervirens* L.) used in this study were purchased from Laguana Moon (https://lagunamoon.com/collections/aromatherapy). Four different concentrations (7.5, 15, 30, and 60%) of each oil were prepared using Tween 80 as an emulsifier. These concentrations were chosen based on a pilot test.

### Preparation of nanoemulsions

The Sugumar et al.^28^ preparation method was modified slightly to produce nanoemulsions using a low-energy approach. Three oils, myrrh, patchouli, and cypress, a non-ionic surfactant (0.5% Tween 80), and distilled water were used to create the oil-in-water nanoemulsion. For all formulations, the oil concentration (20% v/v) remained constant. Tween 80, a non-ionic surfactant, and oil were combined in a 1:1 ratio at room temperature, the liquid was homogenized, and the obtained nanoemulsion was stirred intermittently for three hours over a magnetic stirrer at a high-speed of 2000 rpm then, the resulting nanoemulsion was left stirring overnight.

### Characterization of nanoemulsion

#### Transmission electron microscope (TEM)

A high-resolution transmission electron microscope (HRTEM), JEOL model JEM-2100, was utilized to analyse the particle shape and size of the generated samples. On a copper grid coated with carbon, drops of the diluted formulations were placed, dried at room temperature for 10 min, examined, and recorded nano-size images of the samples.

#### Particle size distribution analysis

The particle size distribution analysis was carried out with a Malvern Zetasizer 3000 HAS using dynamic light scattering (DLS) technique at a run time of 2 min, temperature of 23 °C, using water as a solvent at a concentration of 1 mg/mL. To reduce the effects of multiple scattering, the prepared emulsions were diluted with milli-Q (Millipore corporation) double-distilled water before the experiment. To decrease the particle size, the three nanoemulsions’ ideal formulations were further optimized at the high-speed magnetic stirrer’s speed.

#### Bioassay of oils and nanoemulsions against unfed adult ticks

Two-week-old unfed adults of *R. sanguineus (s.l.)* ticks were tested using an immersion test according to Abdel-Ghany et al.^29^. The used concentrations for oils were 7.5,15,30 and 60% while the concentrations of nanoemulsion were 2.5,5,10 and 20%. In the bioassay experiment, the used concentrations were selected based on a pilot study to detect the suitable concentrations of oils and nanoemulsions against unfed adults of *R. sanguineus* ticks. The pilot test depends on testing folded concentrations starting from the highest one which gave 100% mortality to the lowest one which gave zero mortality. Following this, the concentrations that gave lower and higher than 50% mortalities were chosen for the bioassay experiments. Five ml of each concentration was used for immersion of thirty unfed adults for 2 min and was divided into three replicates after treatment, 10 adults/ replicate. Each replicate has an equal number of males and females (5 males and 5 females). The unfed adults immersed in 5 ml of the emulsifier (Tween 80) for 2 min were considered as a negative control for oils, while unfed adults immersed in 5 ml of distilled water were considered negative control for nanoemulsions. Unfed adults immersed in Deltamethrin (1 ml/L) for 2 min were considered as reference acaricide. After immersion, treated adults were transferred to filter paper and then placed in plastic cups, incubated, and followed for 7 days to observe and record the mortality in ticks.

### In vitro cytotoxicity study using MTT assay

#### Cell culture

Normal fibroblast cells (BJ-1) were maintained in DMEMF12 supplemented with 10% fetal bovine serum. Cells were incubated at 37 °C with 5% CO2 and 95% humidity. Trypsin 0.15% was used to subculture the cells. Professor Stig Linder, Oncology and Pathology department, Karolinska Institute, Stockholm, Sweden, kindly provided the skin normal human cell line (BJ-1) immortalized normal foreskin fibroblast cell line.

#### Cell viability assay

The medium was changed to serum-free 24 h after seeding 50, 000 cells per well. The cells were subsequently exposed to myrrh, patchouli, and cypress oils at various concentrations of 0.63, 1.25, 2.5 and 5%. The cells were also exposed to 0.08, 0.16, 0.31, 0.625, and 1.25% of myrrh, patchouli, and cypress oil nanoemulsion. Different concentrations of tested substances were used to determine the safe concentration on skin cells that show less toxicity on the cells and to determine safe doses when used. The assay was performed in triplicates. The cells were treated for 48 h. Cell viability was measured using the MTT (3-(4, 5-dimethylthiazol-2-yl)-2, 5-diphenyl tetrazolium bromide) assay as described by Mosmann^30^. The equation used for the calculation of percentage cytotoxicity: (1- (av(x) / (av (NC)) * 100.

Where Av: average, X: absorbance of the sample well measured at 595 nm with reference 690 nm, NC: absorbance of negative control measured at 595 nm with reference 690.

#### Statistical analysis

One-way ANOVA followed by post hoc the Tukey and survival time using Kaplan–Meier analysis were used to statistically analyse the data using the SPSS program version 22 (IBM Corp., Armonk, NY). Regression equation analysis was used to determine the LC_50_ and LC_90_ values using the mortality probit-transformed data. Using Ehab software, the probit method^31^ was used to analyse the dose-response data.

#### Ethics Declaration

All procedures were designed following the ARRIVE guidelines (PLoS Bio 8(6), e1000412, 2010​) and conducted following the eighth edition (2011) of the rules for the care and use of laboratory animals. They were all authorized by the Ethical Committee for Medical and Veterinary Research at the National Research Centre (NRC), Egypt in accordance with local laws and regulations with serial number 24,712,012,023.

## Results

### Characterization of the prepared nanoemulsions

**Fig. 1:**
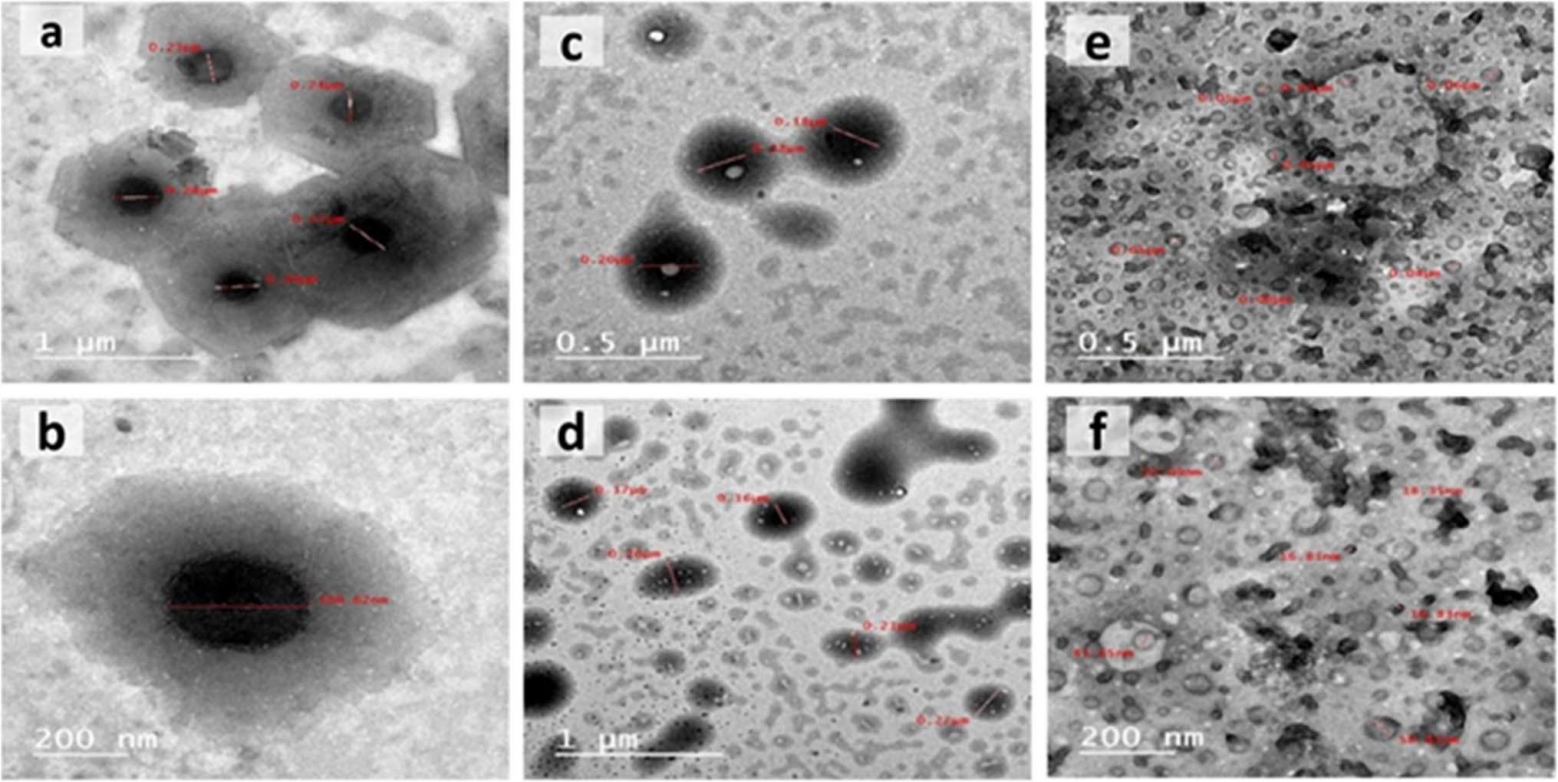
Transmission electron microscopy (TEM) image of oil nanoemulsions showing the size of some oil droplets. (**a **& **b**) myrrh oil nanoemulsion. (**c **& **d**) patchouli oil nanoemulsion. (**e **& **f**) cypress oil nanoemulsion.

**Fig. 2:**
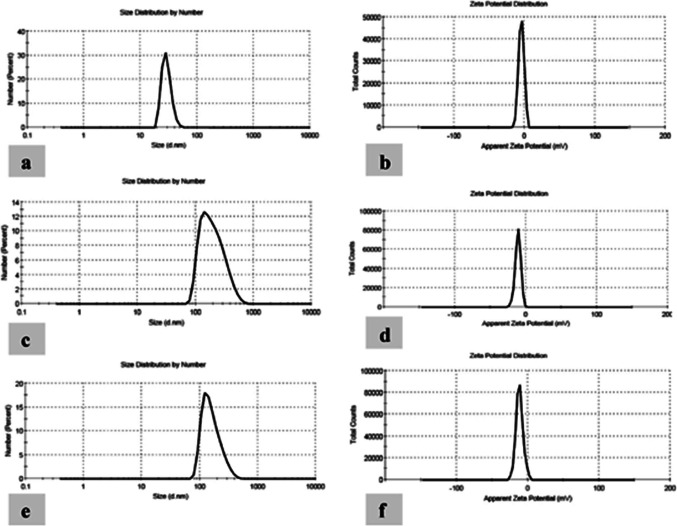
Droplet size distribution and zeta potential of nanoemulsion. (**a **& **b**) myrrh oil nanoemulsion. (**c** & **d**) patchouli oil nanoemulsion. (**e **& **f**) cypress oil nanoemulsion.

According to TEM evaluation, the particle size of the tested nanoemulsions oils varied from 29 to 211 nm and the droplets were spherical in nature (Fig. 1). The spherical oil droplets were monodispersed with uniform particle size, confirming the particle size which was determined using particle size distribution analysis as shown in (Fig. 2).

Zeta potential can be used to measure the state of the surface of particle size and prediction of long-term stability. Results recorded that the particle size (nm), zeta potential (ZP), and polydispersity index (PDI) for all nanoemulsion formulations decreased the particle size and were narrowly distributed. The droplet size of myrrh NE was 29.30 nm with PDI 0.367. For patchouli, the droplet size was 211.6 nm with PDI 0.221. The cypress droplet size was 164.2 nm with PDI 0.237 (Table 1).

**Table 1:** Droplet size, polydispersity index, and Zeta potential of the nanoemulsion prepared with myrrh, patchouli, and cypress oils.

Nanoemulsion	Particle size (nm)	Zeta potential (mV)	Polydispersity index
Myrrh	29.30	-4.23 ± 3.87	0.367
Patchouli	211.6	-11.1 ± 3.94	0.221
Cypress	164.2	-11.6 ± 5.04	0.237

### Anti-tick effect of oils and nanoemulsions against unfed adult ticks

**Fig. 3:**
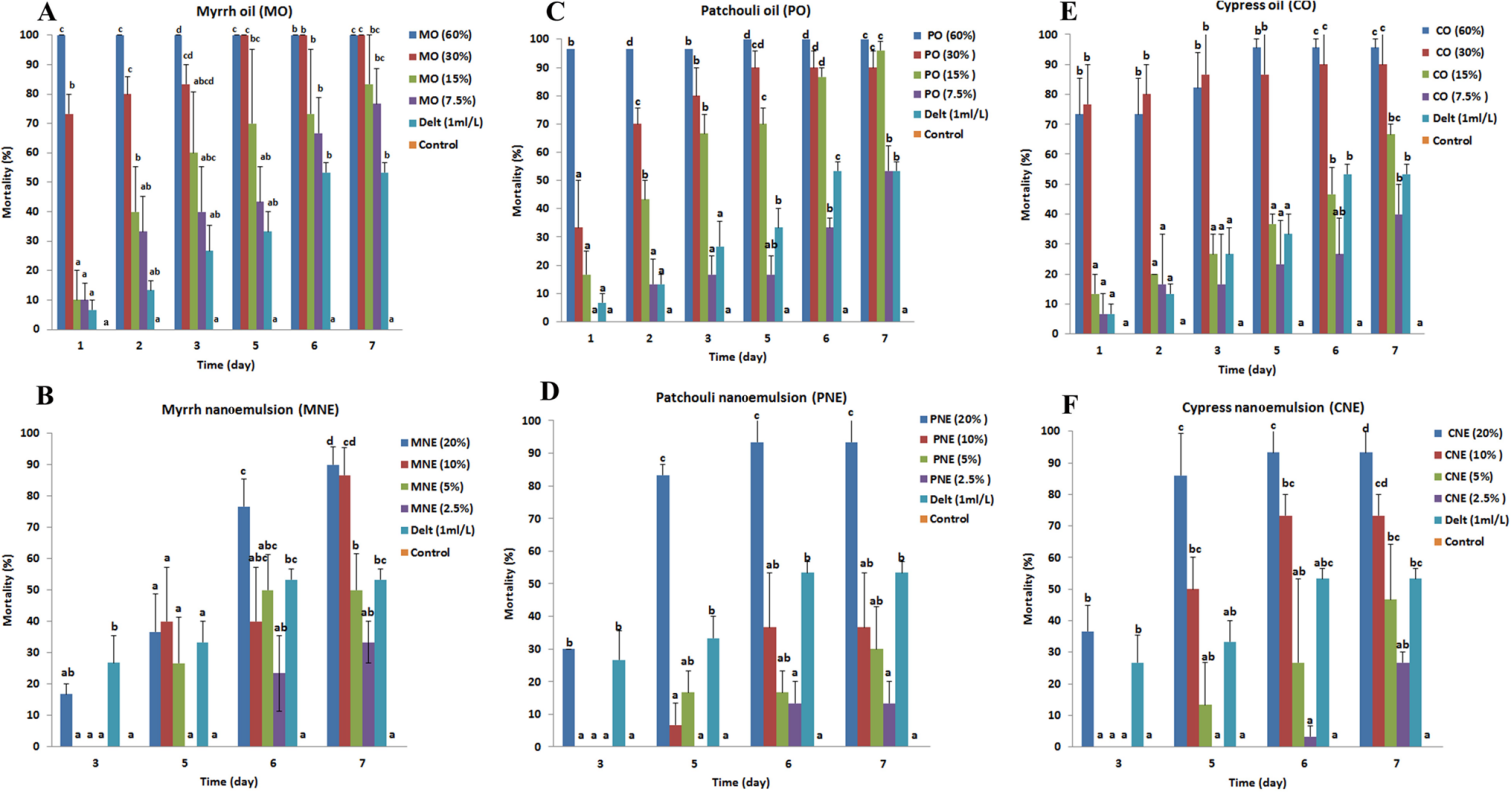
Mortality percentages of *Rhipicephalus sanguineus sensu lato* unfed adults treated with different oils and their nanoemulsions throughout 7 days; **A**: Myrrh oil (MO), **B**: Myrrh nanoemulsion (MNE), **C**: Patchouli oil (PO), **D**: Patchouli nanoemulsion (PNE), **E**: Cypress oil (CO), **F**: Cypress nanoemulsion (CNE).Delt: Deltamethrin.

Figure 3 presents the acaricidal activity of the three tested oils and their nanoemulsions against unfed adults of *R. sanguineus (s.l.)*. It was observed that the effect of tested oils either in their ordinary form or in the NEs form were concentration and time-dependent and the time factor was crucial in increasing the mortality percentage with a significant difference. The results indicated that the tested materials revealed a significant effect on *R. sanguineus (s.l.)* unfed adults compared with reference acaricide (Deltamethrin ml/L). Negative controls either for oils (Tween 80) or nanoemulsion (distilled water) didn’t record any mortality for unfed adults.

Myrrh oil in its ordinary form recorded 100% mortality within 24 h of treatment at a concentration of 60% meanwhile, the lowest concentration of 7.5% recorded 76.6% mortality after 7 days of its application. Myrrh in NE form was dependent on time where it recorded 90% at its highest concentration (20%) and 33.3% mortality at its lowest concentration (2.5%) after 7 days of application (Fig. 3A, B).

Patchouli oil gave 96.6% mortality at the highest concentration after 24 h and reached 100% after 3 days of application while the lowest concentration (7.5%) showed higher efficacy after 7 days (53.3% mortality). Patchouli NE resulted in 93.3% and 13% mortality at a concentration of 20 and 2.5% after 7 days of treatment (Fig. 3C, D).

Cypress oil in ordinary form gave a mortality rate lower than myrrh and Patchouli oil after 24 h where 73.3% mortality was achieved at a concentration of 60%. This percentage increased to 95.5% within 7 days of treatment. Moreover, at a concentration of 7.5% the mortality reached 40% within 7 days of treatment. Cypress NE at a higher concentration of 20% gave 36.6% mortality. This percentage increased on day 7 after treatment to 93.3% (Fig. 3E, F).

**Fig. 4:**
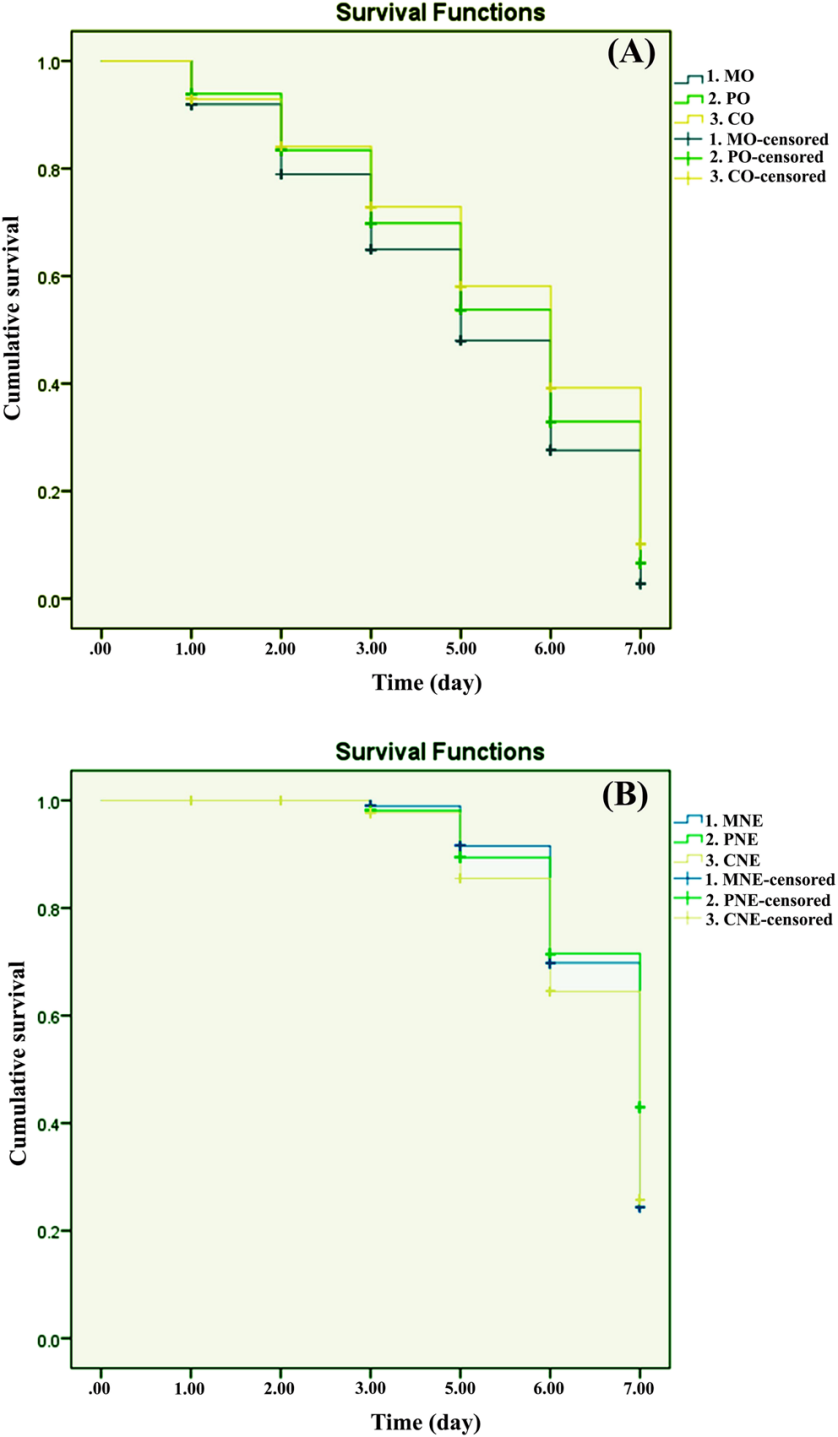
Kaplan–Meier analysis of cumulative survival of *Rhipicephalus sanguineus* adults exposed to three oils (**A**): (1) Myrrh oil (MO), (2) Patchouli oil (PO), (3) Cypress oil (CO) and their nanoemulsions (**B**): (1) Myrrh nanemulsion (MNE), (2) Patchouli nanoemulsion (PNE), (3) Cypress nanoemulsion (CNE).

Figure 4 shows Kaplan-Meier survival curves for the three tested oils (Fig. 4A) and their nanoemulsions (Fig. 4B) on the unfed adults of *R. sanguineus*. The survival curves showed the influence of either oils or their nanoemulsions exposure on the tick survival. The distributions of tick survival were significantly different between the oils exposure groups (*P* < 0.01) and their nanoemulsions groups (*P* < 0.05).

The calculated LC_50_ and LC_90_ for myrrh, patchouli, and cypress oils after 5 days of treatment were (9.01 & 20.72%), (12.40 & 26.59%), and (15.21 & 40.30%), respectively (Table 2). Moreover, the calculated LC_50_ and LC_90_ for myrrh, patchouli, and cypress NEs on 7 days after treatment were (4.17&16.73%), (8.57 & 28.07%), and (5.04 &18.28%), respectively (Table 2). Thus, LC_50_ and LC_90_ findings implay that myrrh EO in its ordinary form exhibited higher acaricidal activity followed by patchouli and cypress. However, In NE forms, myrrh was more toxic followed by cypress and patchouli NEs.

**Table 2:** LC_50_ and LC_90_ of oils 5 days after treatment and their nanoemulsions 7 days after treatment.

Treatments	LC_50_ (%)	LC_90_ (%)	Slope ± SE
Myrrh oil	9.01	20.72	0.41 ± 3.54
Myrrh nanoemulsion	4.17	16.73	0.22 ± 2.12
Patchouli oil	12.40	26.59	0.35 ± 3.86
Patchouli nanoemulsion	8.57	28.07	0.23 ± 2.48
Cypress oil	15.21	40.30	0.26 ± 3.028
Cypress nanoemulsion	5.04	18.28	0.22 ± 2.29

LC_50_: Lethal concentration for 50% of individuals, LC_90_: Lethal concentration for 90% of individuals.

### In vitro cytotoxicity assay

**Fig. 5:**
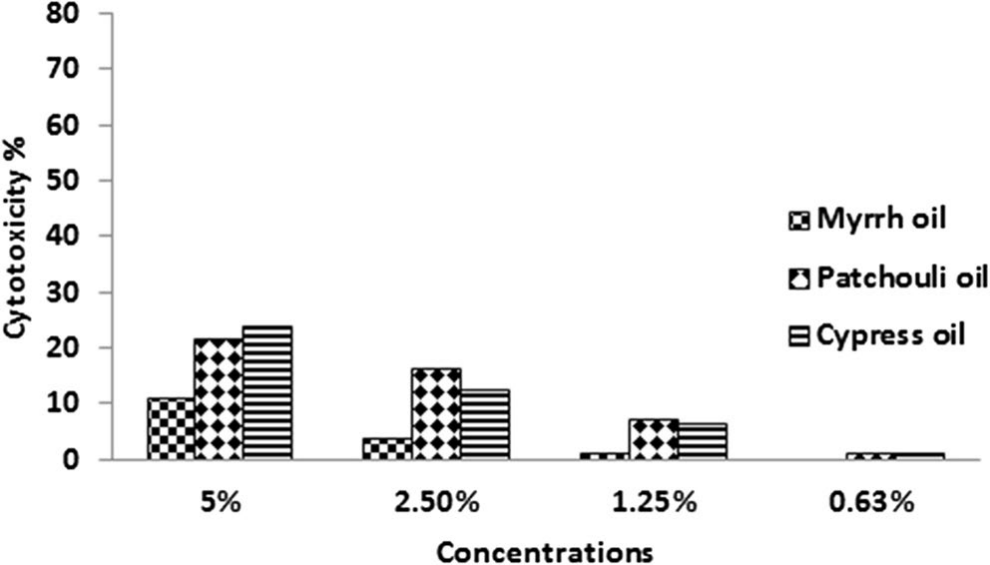
Cytotoxic activity of myrrh, patchouli, and cypress oils against BJ-1 cells determined by MTT assay. Cells were exposed to different concentrations of the samples for 48 h.

**Fig. 6:**
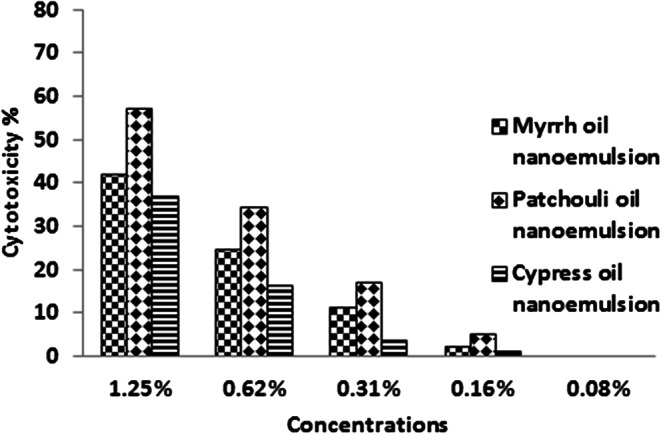
Cytotoxic activity of myrrh, patchouli, and cypress nanoemulsion against BJ-1 cells determined by MTT assay. Cells were exposed to different concentrations of the samples for 48 h.

The normal fibroblast cells were used to determine the safety of the three oils and their prepared nanoemulsions. It was observed that of the three oils at 5% had low cytotoxicity of 10.6, 21.5, and 23.6% for myrrh, patchouli, and cypress, respectively against normal fibroblast cells (Fig. 5). The results of nanoemulsion indicated these compounds were recorded to be safe for normal cells up to a concentration of 0.62% which recorded cytotoxicity% 24.4, 34.4, and 16.3% for myrrh, patchouli, and cypress, respectively. Moreover, at a concentration of 1.25%, a moderate cytotoxic effect appeared for the nanoemulsion of myrrh (42.2%), patchouli (57.0%), and cypress (36.8%) (Fig. 6).

## Discussion

Owing to the need for the development of new and safe alternatives to chemical acaricides for tick control, researchers tried to use phytochemicals to be incorporated into the control program^32,33^. Oils derived from medicinal and aromatic plants contain several compounds which individually or together have promising acaricidal activity. This study revealed that the acaricidal activity of the tested materials against unfed adults of *R. sanguineus (s.l.)* was increased significantly with increased concentration and exposure time. This was in accordance with the study of Gebre-Egziabiher^34^ that revealed the botanical effect was dose concentration- and exposure time-dependent. The results of the present study showed that the three oils and their nanoemulsion have significant acaricidal activity against unfed adults of *R. sanguineus (s.l.)* compared with reference acaricide (Deltamethrin 1 ml/L). To our knowledge, this is the first report testing the acaricidal efficacy of these oils and their NEs against unfed adults of *R. sanguineus (s.l.).* However, only cypress oil was used as acaricide against the tick *Hyalomma scupense*^25^.

In the current study, myrrh oil in the ordinary form recorded 100% mortality for *R. sanguineus* unfed adults comparable with 6.67% mortality for deltamethrin 24 h after treatment. This oil was previously shown a repellency effect against the mite pests such as *Tetranychus urticae* Koch (Acari: Tetranychidae)^22^, the efficiency attributed to the penetration of the oil into the tegument layers. Additionally, it showed insecticidal activity against the insect pest *Spodoptera littoralis boisd* (Boisduval, 1833) (lepidoptera: Noctuidae) 7 days after its application at a concentration of 10% which resulted in 44.4% mortality^35^.

Regarding patchouli oil, it showed 96.6% activity against unfed adults of *R. sanguineus (s.l.)* 24 h after treatment at a concentration of 60% this concentration was comparable to Trongtokit et al.^36^ where 50% patchouli oil gave repellant protection against mosquitoes species (60 min against; *Aedes aegypti* (Linnaeus *in* Hasselquist, 1762), 120 min against *Culex quinquefasciatus* Say, 1823 and 150 min against *Anopheles dirus* Peyton & Harrison, 1979). Moreover, patchouli essential oil showed larvicidal and repellent activity against *Aedes aegypti*^37^ and repellant activity against urban ants (Hymenoptera: Formicidae)^38^. Feng et al.^39^ evaluated the toxic and repellant effects of patchouli essential oil against stored product insects such as *Liposcelis bostrychophila* Badonnel, 1931 (Psocoptera: Liposcelididae), the red flour beetles *Tribolium castaneum* (Herbst, 1797) (Coleoptera: Tenebrionidae), and the cigarette beetles *Lasioderma serricorne* (Fabricius, 1792) (Coleoptera: Anobiidae).

Regarding cypress oil, it exhibited a latent effect against unfed adults of *R. sanguineus (s.l.)* where mortality reached 95.5% within five days after treatment at a concentration of 60%. This oil expressed 100% and 94.4% toxicity against *Hyalomma scupense* engorged females and larvae at a concentration of 20 mg/ml in the study conducted by Alimi et al.^25^ who returned the toxic effect to the AChE inhibition. Other studies highlighted that cypress oil had both good toxicity and repellency against mosquitoes such as *Aedes albopictus* (Skuse, 1894) and *Culex quinquefasciatus* Say^40,41^. Furthermore, it exhibited an insecticidal effect against the maize weevil *Sitophilus zeamais*^42^ (Motschulsky), 1855 and the rice weevil, *Sitophilus oryzae*^43^ (Linnaeus, 1763).

Comparing the data present in the study and considering that myrrh and patchouli oil resulted in a rapid and higher mortality rate against *R. sanguineus (s.l.)* unfed adults than the cypress oil. Moreover, the three tested oils gave significantly higher mortalities than the reference acaricide Deltamethrin. This may be attributed to the presence of more than one active ingredient in the oil that acts synergistically and potentiates the activity, impeding the formation of resistance unlike chemical acaricides.

In this study, a relatively high concentration of oils was used in comparison with previous works. One reason is that other work was conducted on the immature stages (larvae and nymphs) which appear to be more susceptible to acaricides than the most resistant mature stage. In the previous literature, the higher effect of the oils on larvae than adults used in this study might be related to the cuticular thickness that is thicker in adults than larvae and cuticular respiration occurred in the larvae^44^. This hypothesis is supported by different previous literature. Oliveira et al.^45^ found that 100,000 µg. mL^−1^ of *Ocimum gratissimum* essential oil gave no mortality against *R. sanguineus (s.l.)* adults and 81% mortality on nymphs, meanwhile 60,000 µg. mL^−1^ concentration gave 94% mortality on larvae. Rey-Valeiron et al.^46^ evaluated the activity of *Schinus molle* essential oil against the larvae and adult *R. sanguineus* tick where 99.3% mortality was recorded for larvae at a concentration of 2% while engorged adult females recorded no mortality at 20% despite affecting their reproductive efficiency.

According to TEM evaluation to study the surface of the NEs, the particle size ranges from 29 to 211 nm and the droplets were monodispersed and spherical in nature. The droplet size distribution analysis and polydispersity index (PDI) of myrrh, patchouli, and cypress oil NE formulations were determined. The PDI is a degree of homogeneity and stability of the droplet size in the NE system, herein, the PDI of the tested nanoemulsions ranged from 0.2 to 0.3. This is following Harun et al.^47^ who stated that PDI values from zero to one indicate a narrow size distribution and thus provide long-term stability to the formulated NEs. When an oil phase is dispersed in an aqueous phase, the emulsion is stabilized by the presence of a surfactant that forms a layer at the droplet interface, separating the oil from the aqueous phase due to the emulsion’s stability^48^. This separation of the oil from the aqueous phase results in the formation of NEs. The attractive forces between the droplets are proportional to their size, so when the droplet size is in the nano range, the attractive forces between the droplets are weak, preventing particle aggregation and making the NEs more stable^49,50^.

Herein, the acaricidal activity of NEs against unfed adults of *R. sanguineus (s.l.)* tick had a latent effect compared to the oil in an ordinary form where the effect of NE began to appear 72 h after treatment. The three oils in their ordinary form acted quicker to produce their effect on unfed adults of *R. sanguineus (s.l.)* as compared to NEs form. This was in accordance with Rocha et al.^51^ who tested *Pogostemon cablin* (Patchouli) essential oil and its NE against leaf-cutting ants *Atta opaciceps* (Borgmeier, 1939), *Atta sexdens* (Linnaeus, 1758) where essential oil acted more quickly than NEs. Cypress NE produces a higher effect than in ordinary form. These finding matches well with Almadiy and Nena^40^ who stated that cypress NE had a better effect against *Culex quinquefasciatus* than the ordinary oil.

It was previously shown that the small size of the NE particles increases their contact surface and consequently enhances the absorption and have higher mobility with a gradual release leading to increased systemic activity^52,53^. As well nanoformulation improves the stability of the oils and minimizes the used concentrations. Little work was performed using NE of the myrrh, patchouli, and cypress oil either on ticks or other insects. One study evaluated cypress NE as larvicidal and adulticidal activity against *Culex quinquefasciatus* where the recorded mortality was 100% for both adults and larvae at a concentration of 40 µg/ml and 20.0 µl/l respectively^40^.

In the present study, although the effect of NEs on the *R. sanguineus* adults was delayed and produced its higher effect after 7 days of treatment which was near 95%, it provides the advantage of using a lower concentration than that used in the oil which may appear to be economic, where it becomes necessary to obtain an acaricide with good effectiveness and low costs.

As an alternative for lab animals, we applied the cytotoxicity of myrrh, patchouli, and cypress on normal fibroblast cells for the first time. The three oils at 5% had low cytotoxicity of 10.6, 21.5, and 23.6% for myrrh, patchouli, and cypress, respectively against normal fibroblast cells. The results of nanoemulsion indicated these compounds were revealed to be safe for normal cells up to a concentration of 0.62%. The results demonstrate a cytotoxic effect of nanoemulsions at lower concentrations compared with higher concentrations of oils. This returns to the surfactant used in the preparation of nanoemulsion. These results were in accordance with Milhomem-Paixão et al.^54^ who tested the cytotoxic effect of Andiroba oil (AO) and Andiroba nanoemulsion (AN) on fibroblast cell lineage NIH/3T3. These results demonstrate a cytotoxic effect of the AO, at the highest concentrations, and of the surfactant used in producing the nanoemulsion. So, further studies may be needed to determine the action of these formulations using different cell lines.

## Conclusion

Myrrh, patchouli, and cypress oils and their nanoemulsions revealed good anti-tick efficacy against unfed adults of the brown dog tick *Rhipicephalus sanguineus (s.l.).* From the calculated LC_50_ and LC_90_, Myrrh oil in its ordinary form exhibited higher acaricidal activity followed by patchouli and cypress. In nanoemulsion forms, myrrh was more toxic followed by cypress and patchouli NEs. Furthermore, normal fibroblast cells were used as an alternative for lab animals to measure the cytotoxicity of myrrh, patchouli, and cypress. In the next work, in vivo studies in laboratory animals and the cost/benefit ratio should be considered so that can correctly judge the application of these used materials and ensure their use in the field for tick control.

## Supplementary Information

Supplementary Material 1.

Supplementary Material 2.

## Data Availability

All data generated or analyzed during this study are either included in this published and its supplementary materials file (or available from the corresponding author upon reasonable request). The three oils; myrrh, patchouli, and cypress were purchased from Laguna Moon (https://lagunamoon.com/collections/aromatherapy).
