# Supplementary material for: Comparative toxicity of three variant oils and their nanoemulsions on the brown dog tick Rhipicephalus sanguineus
Source: Sci Rep. 2024 Nov 7;14:27060. doi: 10.1038/s41598-024-77402-w (PMC11543673; doi:10.1038/s41598-024-77402-w)
Supplement: Supplementary file 1 — Supplementary Material 1. [file 41598_2024_77402_MOESM1_ESM.docx]

**Table S1.** Gas chromatography-mass spectrometry of Myrrh oil

| Peak | RT | Name | Formula | Area | Area % |
| --- | --- | --- | --- | --- | --- |
| 1 | 8.83 | Alpha-Pinene | C10H16 | 407257.11 | 0.72 |
| 2 | 9.968 | Beta-Pinene | C10H16 | 511559.45 | 0.91 |
| 3 | 10.254 | Beta-Myrcene | C10H16 | 230016.35 | 0.41 |
| 4 | 10.621 | Alpha-Phellandrene | C10H16 | 294858.28 | 0.52 |
| 5 | 10.702 | Beta-Ocimene | C10H16 | 1105049 | 1.96 |
| 6 | 10.93 | Alpha-Terpinene | C10H16 | 382836.81 | 0.68 |
| 7 | 11.178 | Limonene | C10H16 | 2096586.36 | 3.72 |
| 8 | 11.282 | O-Cymene | C10H14 | 1965952.17 | 3.49 |
| 9 | 11.425 | Eucalyptol | C10H18O | 507608.75 | 0.9 |
| 10 | 11.868 | Gamma-Terpinene | C10H16 | 180434.04 | 0.32 |
| 11 | 12.549 | ALPHA-TERPINOLENE | C10H16 | 1278902.69 | 2.27 |
| 12 | 13.473 | Linalool | C10H18O | 200798.43 | 0.36 |
| 13 | 15.687 | Methyl salicylate | C8H8O3 | 10128786.36 | 17.97 |
| 14 | 19.897 | Aromandendrene | C15H24 | 9052907.01 | 16.07 |
| 15 | 20.211 | Farnesol | C15H26O | 3029742.25 | 5.38 |
| 16 | 20.43 | Farnesol | C15H26O | 2864226.21 | 5.08 |
| 17 | 21.035 | Trans-Caryophyllene | C15H24 | 612750.61 | 1.09 |
| 18 | 21.168 | Alpha-Bulnesene | C15H24 | 4704759.57 | 8.35 |
| 19 | 22.635 | Alpha-Humulene | C15H24 | 474669.65 | 0.84 |
| 20 | 24.545 | Patchouli alcohol | C15H26O | 4065839.85 | 7.22 |
| 21 | 26.321 | Isopropyl myristate | C17H34O2 | 12255625.76 | 21.75 |

RT: Retention Time

**Table S2**. Gas chromatography-mass spectrometry of Patchouli oil

| Peak | RT | Name | Formula | Area | Area % |
| --- | --- | --- | --- | --- | --- |
| 1 | 19.897 | Aromandendrene | C15H24 | 7402278.56 | 14.76 |
| 2 | 20.083 | Tridecane, 3-ethyl- | C15H32 | 1048674.41 | 2.09 |
| 3 | 20.431 | Farnesol | C15H26O | 3044334.88 | 6.07 |
| 4 | 21.173 | Alpha-Bulnesene | C15H24 | 6004550.37 | 11.97 |
| 5 | 24.545 | Patchouli alcohol | C15H26O | 11385199.42 | 22.71 |
| 6 | 26.326 | Isopropyl myristate | C17H34O2 | 21257844.08 | 42.39 |

RT: Retention Time

**Table S3** Gas chromatography-mass spectrometry of Cypress oil

| Peak | RT | Name | Formula | Area | Area Sum % |
| --- | --- | --- | --- | --- | --- |
| 1 | 20.507 | Trans-Caryophyllene | C15H24 | 197685.5 | 4.15 |
| 2 | 20.597 | Farnesol | C15H26O | 696336.35 | 14.61 |
| 3 | 20.745 | Benzene, 1-(1,5-dimethyl-4-hexenyl)-4-methyl- | C15H22 | 475914.62 | 9.99 |
| 4 | 20.983 | Alpha-Muurolene | C15H24 | 792643.04 | 16.63 |
| 5 | 21.092 | Trans-Caryophyllene | C15H24 | 554546.46 | 11.64 |
| 6 | 21.254 | Gamma-Elemene | C15H24 | 697874.5 | 14.64 |
| 7 | 21.397 | Delta-Cadinene | C15H24 | 1254930.3 | 26.33 |
| 8 | 22.045 | Alpha-Calacorene | C15H20 | 95703.91 | 2.01 |

RT: Retention Time

**Table S4.** Mortality percentages (Mean±SE) of *Rhipicephalus sanguineus sensu lato* unfed adults treated with myrrh, patchouli and cypress oils.

| Oils | Concentration (%) | Day 1 | Day 2 | Day 3 | Day 5 | Day 6 | Day 7 |
| --- | --- | --- | --- | --- | --- | --- | --- |
| Myrrh | 60 | 100.00±0.00^c^ | 100.00±0.00^c^ | 100.00±0.00^d^ | 100.00±0.00^c^ | 100.00±0.00^b^ | 100.00±0.00^c^ |
|  | 30 | 73.33±6.66^b^ | 80.00±5.77^c^ | 83.33±6.66^cd^ | 100.00±0.00^c^ | 100.00±0.00^b^ | 100.00±0.00^c^ |
|  | 15 | 10.00±10.00^a^ | 40.00±15.27^b^ | 60.00±20.81^bcd^ | 70.00±25.16^bc^ | 73.33±21.85^b^ | 83.33±16.66^bc^ |
|  | 7.5 | 10.00±5.77^a^ | 33.33±12.01^ab^ | 40.00±15.27^abc^ | 43.33±12.01^ab^ | 66.66±12.01^b^ | 76.66±12.01^bc^ |
|  | Deltamethrin (1ml/L) | 6.67±3.33^a^ | 13.33±3.33^ab^ | 26.67±8.82^ab^ | 33.33±6.67^ab^ | 53.33±3.33^b^ | 53.33±3.33^b^ |
|  | Control | 0.00±0.00^a^ | 0.00±0.00^a^ | 0.00±0.00^a^ | 0.00±0.00^a^ | 0.00±0.00^a^ | 0.00±0.00^a^ |
|  | P value | <0.001 | <0.001 | <0.001 | <0.001 | <0.001 | <0.001 |
| Patchouli | 60 | 96.66±3.33^b^ | 96.66±3.33^d^ | 96.66±3.33^b^ | 100.00±0.00^d^ | 100.00±0.00^d^ | 100.00±0.00^c^ |
|  | 30 | 33.33±16.66^a^ | 70.00±5.77^c^ | 80.00±10.00^b^ | 90.00±5.77^cd^ | 90.00±5.77^d^ | 90.00±5.77^c^ |
|  | 15 | 16.66±8.31^a^ | 43.33±6.66^b^ | 66.66±6.66^b^ | 70.00±5.77^c^ | 86.66±3.33^d^ | 96.00±3.33^c^ |
|  | 7.5 | 0.00±0.00^a^ | 13.33±8.81^a^ | 16.66±6.66^a^ | 16.66±6.66^ab^ | 33.33±3.33^b^ | 53.33±8.81^b^ |
|  | Deltamethrin (1ml/L) | 6.67±3.33^a^ | 13.33±3.33^a^ | 26.67±8.82^a^ | 33.33±6.67^b^ | 53.33±3.33^c^ | 53.33±3.33^b^ |
|  | Control | 0.00±0.00^a^ | 0.00±0.00^a^ | 0.00±0.00^a^ | 0.00±0.00^a^ | 0.00±0.00^a^ | 0.00±0.00^a^ |
|  | P value | <0.001 | <0.001 | <0.001 | <0.001 | <0.001 | <0.001 |
| Cypress | 60 | 73.33±12.01^b^ | 73.33±12.01^b^ | 82.22±11.75^b^ | 95.55±2.93^b^ | 95.55±2.93^c^ | 95.55±2.93^c^ |
|  | 30 | 76.66±13.33^b^ | 80.00±10.00^b^ | 86.66±13.33^b^ | 86.66±13.33^b^ | 90.00±10.00^c^ | 90.00±10.00^c^ |
|  | 15 | 13.33±6.66^a^ | 20.00±0.00^a^ | 26.66±6.66^a^ | 36.66±3.33^a^ | 46.66±8.81^b^ | 66.66±3.33^bc^ |
|  | 7.5 | 6.66±6.66^a^ | 16.66±16.66^a^ | 16.66±16.66^a^ | 23.33±14.52^a^ | 26.66±12.01^ab^ | 40.00±10.00^b^ |
|  | Deltamethrin (1ml/L) | 6.67±3.33^a^ | 13.33±3.33^a^ | 26.67±8.82^a^ | 33.33±6.67^a^ | 53.33±3.33^b^ | 53.33±3.33^b^ |
|  | Control | 0.00±0.00^a^ | 0.00±0.00^a^ | 0.00±0.00^a^ | 0.00±0.00^a^ | 0.00±0.00^a^ | 0.00±0.00^a^ |
|  | P value | <0.001 | <0.001 | <0.001 | <0.001 | <0.001 | <0.001 |

a,b…… ect, indicate the significant difference between the means of mortality percentages of ticks according to Tukey test with df= 5,12.

**Table S5.** Mortality percentages (Mean±SE) of *Rhipicephalus sanguineus sensu lato* unfed adults treated with myrrh, patchouli and cypress nanoemulsions.

| Nanoemulsion | Concentration (%) | Day 3 | Day 5 | Day 6 | Day 7 |
| --- | --- | --- | --- | --- | --- |
| Myrrh | 20 | 16.66±3.33^ab^ | 36.66±12.01^a^ | 76.66±8.81^c^ | 90.00±5.77^d^ |
|  | 10 | 0.00±0.00^a^ | 40.00±17.32^a^ | 40.00±17.32^abc^ | 86.66±8.81^cd^ |
|  | 5 | 0.00±0.00^a^ | 26.66±14.52^a^ | 50.00±11.25^abc^ | 50.00±11.54^b^ |
|  | 2.5 | 0.00±0.00^a^ | 0.00±0.00^a^ | 23.33±12.01^ab^ | 33.33±6.66^ab^ |
|  | Control | 0.00±0.00^a^ | 0.00±0.00^a^ | 0.00±0.00^a^ | 0.00±0.00^a^ |
|  | Deltamethrin (1ml/L) | 26.67±8.82^b^ | 33.33±6.67^a^ | 53.33±3.33^bc^ | 53.33±3.33^bc^ |
|  | P value | 0.001 | 0.065 | 0.004 | <0.001 |
| Patchouli | 20 | 30.00±0.00^b^ | 83.33±3.33^c^ | 93.33±6.66^c^ | 93.33±6.66^c^ |
|  | 10 | 0.00±0.00^a^ | 6.66±6.66^a^ | 36.66±16.66^ab^ | 36.66±16.66^ab^ |
|  | 5 | 0.00±0.00^a^ | 16.66±6.66^ab^ | 16.66±6.66^ab^ | 30.00±13.00^ab^ |
|  | 2.5 | 0.00±0.00^a^ | 0.00±0.00^a^ | 13.33±6.66^a^ | 13.33±6.66^a^ |
|  | Control | 0.00±0.00^a^ | 0.00±0.00^a^ | 0.00±0.00^a^ | 0.00±0.00^a^ |
|  | Deltamethrin (1ml/L) | 26.67±8.82^b^ | 33.33±6.67^b^ | 53.33±3.33^b^ | 53.33±3.33^b^ |
|  | P value | <0.001 | <0.001 | <0.001 | <0.001 |
| Cypress | 20 | 36.6±8.31^b^ | 86.00±13.33^c^ | 93.33±6.66^c^ | 93.33±6.66^d^ |
|  | 10 | 0.00±0.00^a^ | 50.00±10.00^bc^ | 73.33±6.66^bc^ | 73.33±6.66^cd^ |
|  | 5 | 0.00±0.00^a^ | 13.33±13.33^ab^ | 26.66±26.66^ab^ | 46.66±17.63^bc^ |
|  | 2.5 | 0.00±0.00^a^ | 0.00±0.00^a^ | 3.33±3.33^a^ | 26.66±3.33^ab^ |
|  | Deltamethrin (1ml/L) | 26.67±8.82^b^ | 33.33±6.67^ab^ | 53.33±3.33^abc^ | 53.33±3.33^bc^ |
|  | Control | 0.00±0.00^a^ | 0.00±0.00^a^ | 0.00±0.00^a^ | 0.00±0.00^a^ |
|  | P value | <0.001 | <0.001 | <0.001 | <0.001 |

a,b…… ect, indicate the significant difference between the means of mortality of ticks percentages according to Tukey test with df= 5,12..
