# Supplementary material for: Comparative toxicity of three variant oils and their nanoemulsions on the brown dog tick Rhipicephalus sanguineus
Source: Sci Rep. 2024 Nov 7;14:27060. doi: 10.1038/s41598-024-77402-w (PMC11543673; doi:10.1038/s41598-024-77402-w)
Supplement: Supplementary file 2 — Supplementary Material 2. [file 41598_2024_77402_MOESM2_ESM.docx]

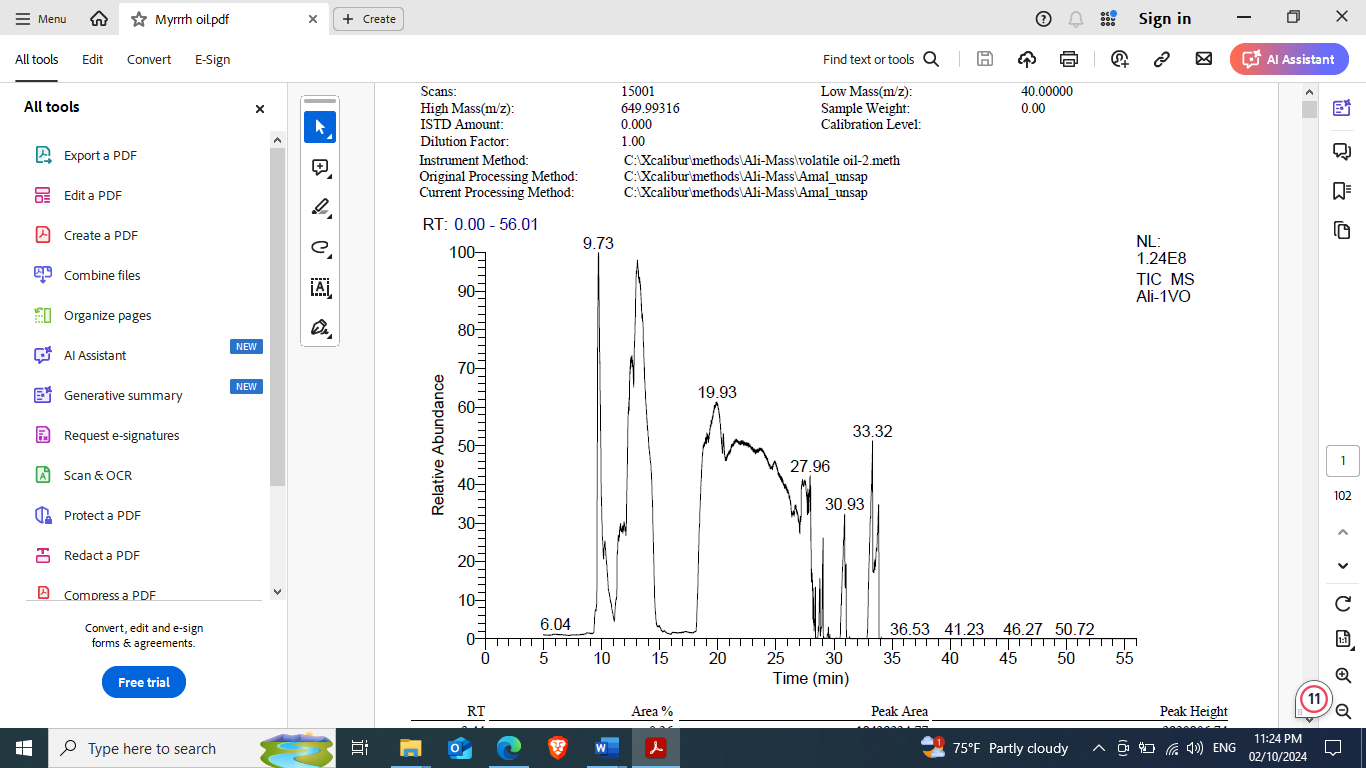


**Figure S1.** Gas chromatogram of the Myrrh oil


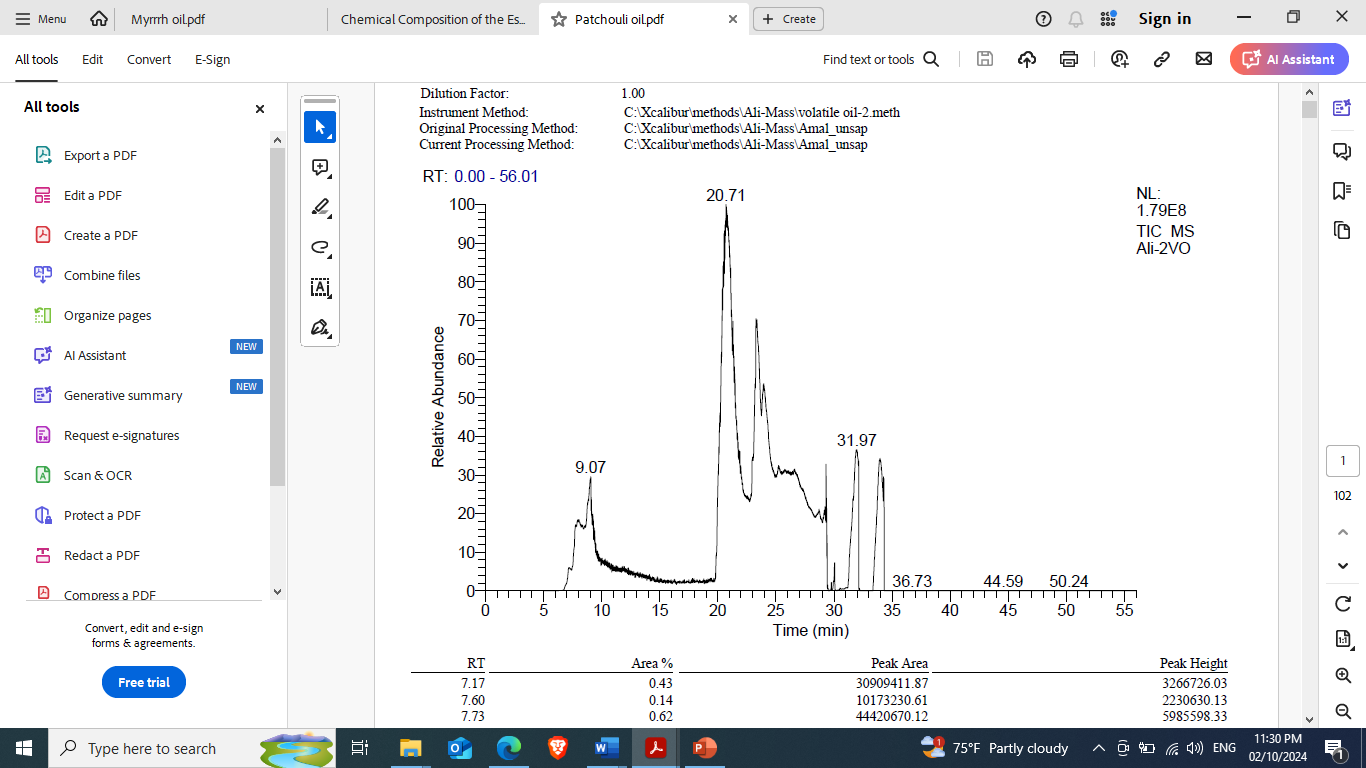


**Figure S2.** Gas chromatogram of the patchouli oil


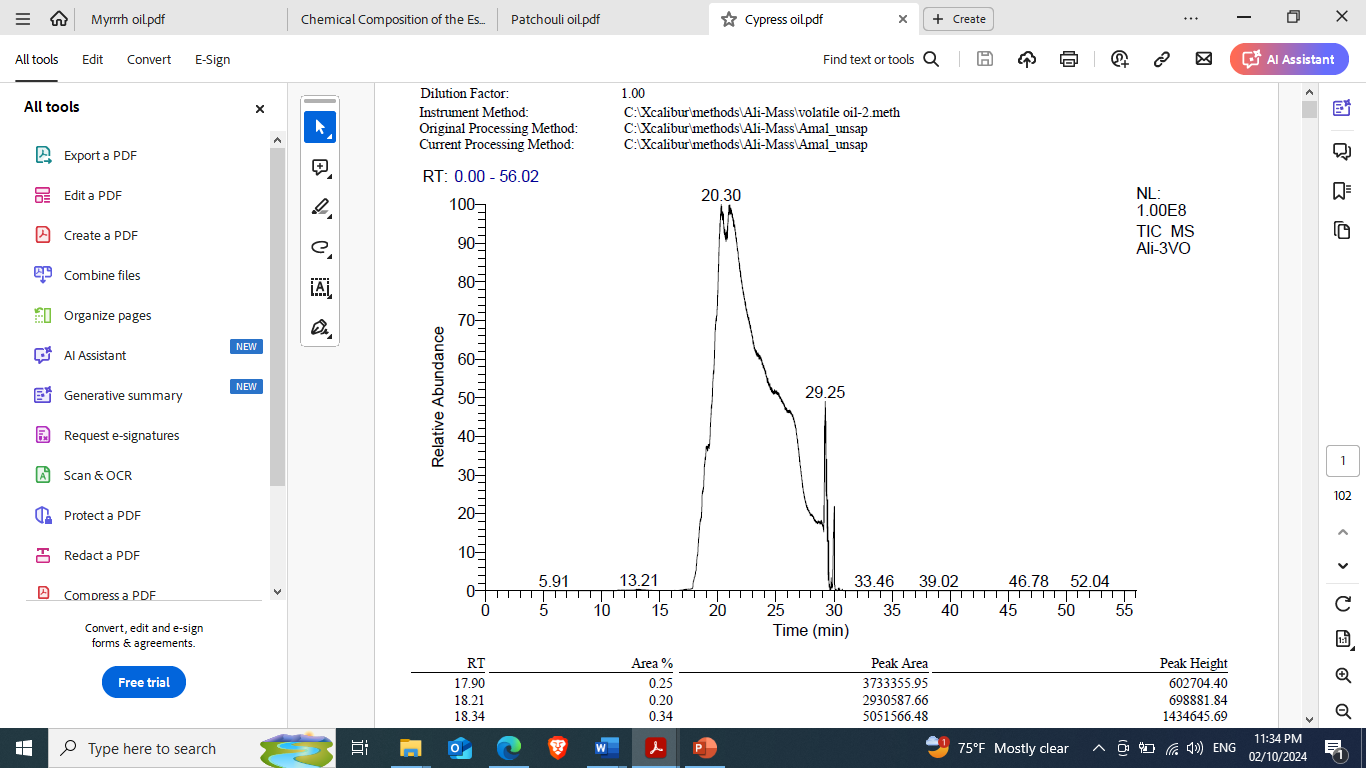


**Figure S3.** Gas chromatogram of the cypress oil
